# Tattoo-Associated Sarcoidosis With Severe Uveitis Successfully Treated With Mycophenolate Mofetil: A Report of Two Cases

**DOI:** 10.7759/cureus.17197

**Published:** 2021-08-15

**Authors:** Nso Nso, Bahtiyar Toz, Tsung Han Ching, Ravali Kondaveeti, Adriana Abrudescu

**Affiliations:** 1 Internal Medicine, Icahn School of Medicine at Mount Sinai, Queens Hospital Center, New York, USA; 2 Rheumatology, Icahn School of Medicine at Mount Sinai, Queens Hospital Center, New York, USA

**Keywords:** tattoo, sarcoidosis, uveitis, case report, mycophenolate mofetil

## Abstract

Tattooing is an increasing trend among Western countries, with about 18% of the population undergoing the procedure once in their lifetime. The process looks simple; introduce exogenous pigment into the dermis layer of the skin, altering the skin color permanently. However, this simple procedure leads to several health issues and medical complications, both acute and chronic, and some are difficult to cure. Sarcoidosis is high on the list of severity involving almost all body organs. Multiple organ involvement makes this condition more difficult to treat. Lungs and lymphatics are the leading sites of involvement, followed by an inflammatory disease of the eye called uveitis. An additional problem is the limited confirmatory diagnostic tests and treatment options for sarcoidosis. Each patient must be considered unique based on their age, clinical presentation, and severity of involvement. Proper treatment must be tailored for better outcomes with minimum side effects and rapid cure. Here we describe two case reports of tattoo-associated sarcoidosis with severe uveitis successfully treated with mycophenolate mofetil.

## Introduction

Sarcoidosis is a multisystem autoimmune granulomatous disease that presents with systemic noncaseating granulomas made up of clusters of different immune cells, including macrophages, mononuclear cells, CD4+ T cells, a few CD8+, and epithelioid cells. Sarcoidosis can affect any body organ, including the skin and eyes [1-4]

The etiology of sarcoidosis is still unknown despite extensive research and effort. Its occurrence differs in different ethnic groups and is 10 times more common in African Americans with a more severe presentation. The predominance in the female population is also slightly higher. African Americans are among the highest in the list with 17-43 annual cases per 100,000 people and a 2:1 female prevalence ratio, Northern Europeans with 11 to 24 cases, Hispanics and Asians with the lowest numbers of one to three per 100,000 people. The peak age group of sarcoidosis is usually between 20 and 39 years of age [5-7].

Tattoo-associated sarcoidosis is rare, and the exact prevalence and incidence of tattoo-associated sarcoidosis is unknown. The term “tattoo sarcoidosis” emerged in the early 1940s, when patients with black tattoos presented with nodules on their tattoos. Further studies reported the increasing inflammatory trend on almost all body sites, including ocular sarcoidosis and uveitis, due to increasing tattoo and permanent makeup trends. Permanent makeup, in addition to tattooing, makes the situation more complex [2].

Approximately one-fourth of patients with sarcoidosis present with ocular involvement, of which 3%-10% exhibit sarcoid/tattoo uveitis with or without systemic sarcoidosis and show bilateral hilar adenopathy or pulmonary reticular opacities [3,7].

Here, we present two cases of tattoo-associated sarcoidosis with pulmonary involvement, uveitis, and cutaneous nodules. Both cases were successfully treated with prednisone and mycophenolate mofetil (MMF).

## Case presentation

Case 1

A 26-year-old Guyanese male who worked at a warehouse presented with worsening left red-eye and blurry vision for two weeks. The ophthalmologist started on prednisolone eye drop, which initially controlled his eye symptoms. However, he noticed new, small skin papules on the left forearm of the tattoo that he had received five years ago. He had no significant medical history or family history of sarcoidosis. On physical examination, he was found to have a bilateral conjunctival injection, a tattoo with small bumps on the left forearm and upper arm, and no erythema or discharge was noted around the tattoo.

Results of laboratory studies, including serum calcium, were normal; additionally, the erythrocyte sedimentation rate (ESR) was 6 mm/h, angiotensin-converting enzyme (ACE) level was 55 U/L (ref 14-82 U/L), and C-reactive protein (CRP) level was 2 mg/L (normal: less than 10 mg/L).

**Figure 1:**
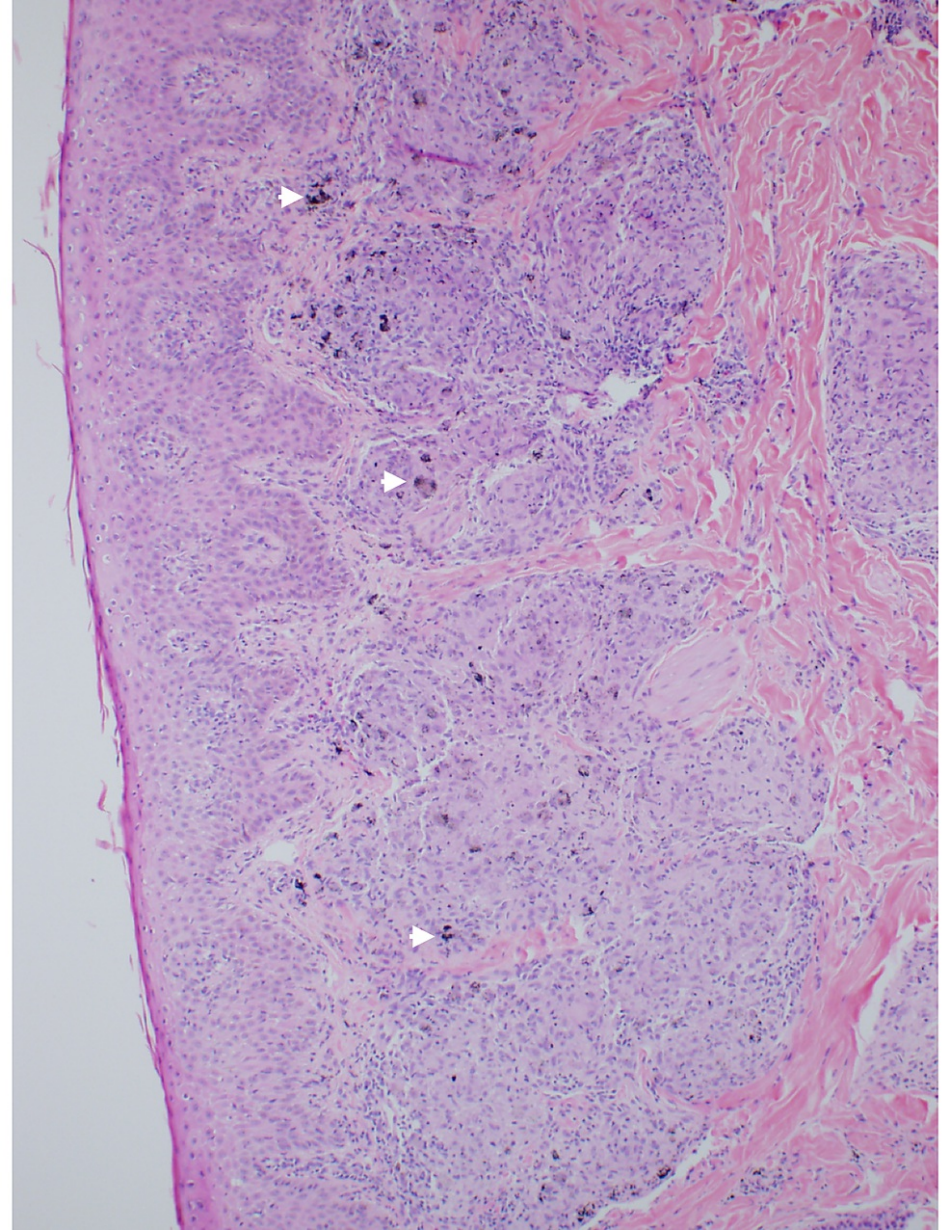
A photomicrograph of a punch biopsy obtained from the papule showing nodular clusters of epithelioid cells occupying the dermis are associated with a sparse inflammatory infiltrate and pigment granules within the papillary dermis (arrowheads) (H&E stain, objective 20x)

Chest X-ray was normal, and chest CT revealed bilateral axillary lymphadenopathy with the largest node noted in the left axillary region measuring 2.1 x 1.2 cm and a 2 mm nodule noted in the right middle lobe. Skin punch biopsy showed the tattoo having sarcoid granuloma (Figure 1).

Tissue culture for mycobacteria and fungi was negative. The patient was started on topical corticosteroid and then mycophenolate mofetil 2 g daily was added to the treatment, as the patient had recurrent uveitis and the patient's symptoms improved after starting mycophenolate mofetil.

Case 2

A 23-year-old Guyanese male presented with left eye pain, which was burning and pricking in nature, and intermittent blurry vision. He received chest tattoos in March 2018, which became inflamed around July 2018. Shortly afterward, the arm tattoos that he received three years ago also became raised. The raised portions were in areas with black and red ink only. On physical examination, he was found to have a bilateral conjunctival injection, and there were raised plaques without erythema within black tattoo margins on the chest. He denied shortness of breath, fevers, chills, cough, or sputum production, and he did not have any significant medical history or family history of sarcoidosis.

**Figure 2:**
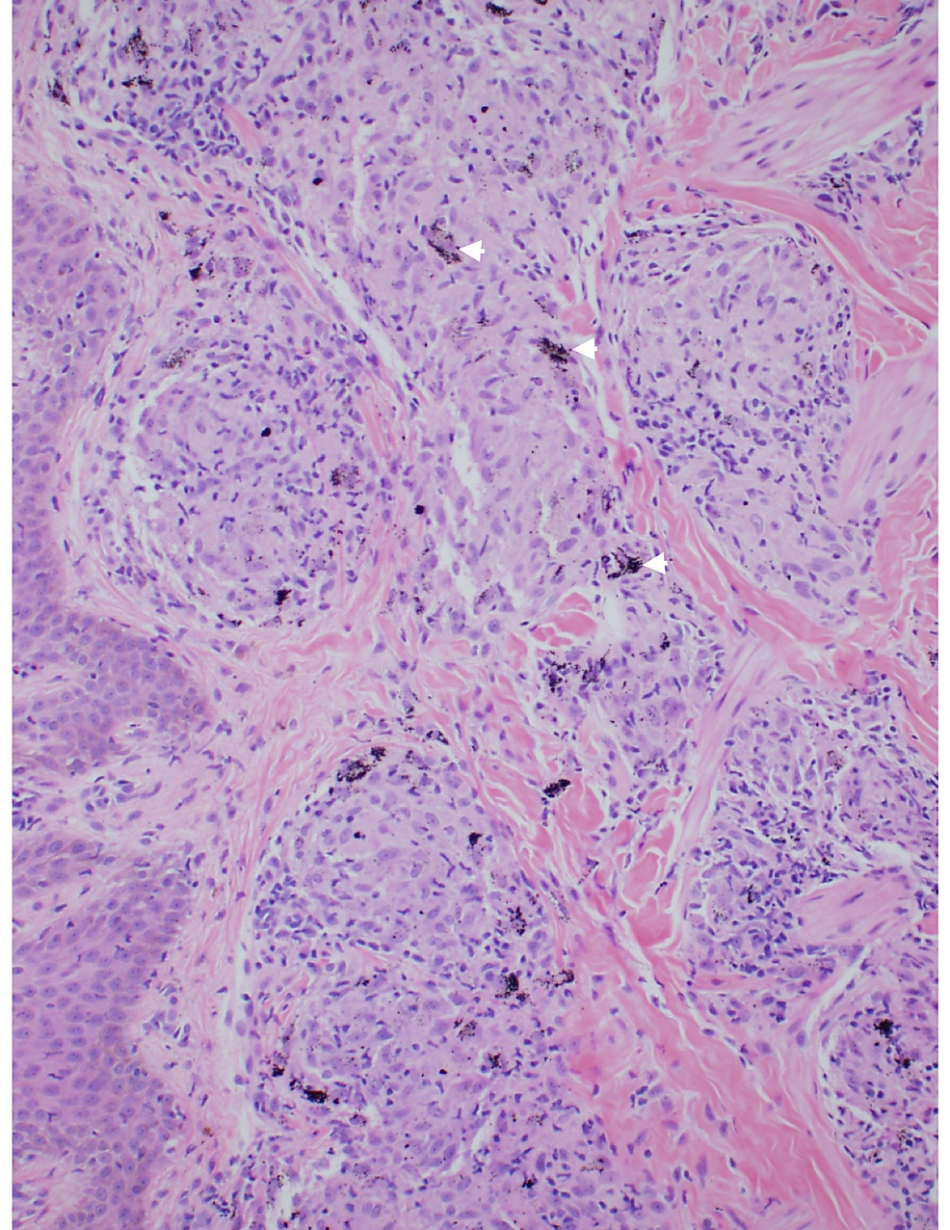
A photomicrograph of a punch biopsy obtained from the papule showing a darkly pigmented material free in the dermis and within macrophages (arrowheads). Nodular clusters of epithelioid cells occupying the dermis are associated with a sparse inflammatory infiltrate) (H&E stain)

Laboratory studies revealed ESR was 24 mm/h with no other additional abnormality. The ACE level was 32 U/L (ref. range 14-82 U/L). Chest CT showed a 2 mm pulmonary nodule in the superior segment of the left lower lobe and a 1 mm nodule in the left upper lobe, which did not show axillary or mediastinal lymphadenopathy. Skin punch biopsy demonstrated a tattoo with non-necrotizing epithelioid granulomas (Figure 2).

Tissue cultures were negative for mycobacteria and fungi. The diagnosis of tattoo-associated systemic sarcoidosis was made on clinical and histological findings. The patient initially was treated with prednisolone 20 mg daily and then started on mycophenolate mofetil 1500 mg daily, as the patient had recurrent uveitis when tapered off prednisolone. After three months of this treatment, the patient's uveitis and skin lesions improved.

## Discussion

Tattoo-associated sarcoidosis with severe uveitis is a condition where inflammation is usually restricted to the tattooed area and the uveal region but lung involvement, including lung nodule and lymphadenopathy, is also reported. This sarcoidosis phenomenon was first published in 1969 with three case reports. Later, this phenomenon was reported by different clinicians with more chronic presentation and limited treatment options [2,8].

Tattoo-associated cutaneous presentations are further divided into specific and nonspecific lesions based on histopathologic features. In the specific form, lesions contain noncaseating granulomas, which are a classic histopathologic finding of sarcoidosis. All other findings are classified as nonspecific. Skin findings may not correlate with the severity of sarcoidosis [9-10]. Both of our patients presented with a specific form of papules and nodules on presentation with severe uveitis. An additional crucial factor is the age group of patients, mostly young to mature adults. Our patients were 26 and 23 years of age.

The next challenge is the definitive diagnosis, as no serum or radiological testing is a true indicator in a case of sarcoidosis. Higher than normal ACE levels may be a sign of sarcoidosis but is not a confirmatory test. ACE levels may be within the normal range with active sarcoidosis, 69% of active cases were reported with normal ACE levels [7,11]. Chest CT identifies pulmonary involvement, and noncaseating granulomas on biopsy reports create a comprehensible picture of inflammation severity, similar to our reports [6].

A relevant history, physical examination, clinical features, laboratory, radiological investigations, and histopathological examination by punch biopsy guide the patient’s condition toward treatment options [12]. The confirmation and final diagnosis of sarcoidosis are still challenging with the limitation of definitive testing parameters available. A punch biopsy is the most crucial test for getting true representation, verification, and severity of the disease. The WHO diagnostic classification (11th revision) described a punch biopsy as a recommended standard [13]. Noncaseating granulomas or sarcoid granulomas in the biopsy report establish the final diagnosis and differentiate between sarcoidosis versus non-sarcoid nodular inflammation [14].

Tattoo sarcoidosis with severe uveitis is usually treated with disease-modifying antirheumatic drugs (DMARDs), and methotrexate is top of the list of clinician prescriptions due to the availability of extensive research, clinical trial data, and authentic literature availability. Other less prescribed DMARDs are azathioprine, leflunomide, mycophenolate, and chloroquine/hydroxychloroquine. Other biological agents are considered as third-line therapeutic options due to higher costs and limited FDA regulations. Anecdotal reports describe the efficacy of MMF in sarcoidosis treatment, recommending the continuous monitoring of CBC and renal function tests every three months [4].

Local treatment of corticosteroid (prednisone) gives positive results in uveitis by suppressing inflammation, pain, and ocular scarring. The therapeutic option chosen for our cases was MMF, which is a successful cytotoxic drug in the case of uveitis. Both of our patients were successfully treated with MMF, labeled as second-line therapy for sarcoidosis. MMF was reportedly well-tolerated in the majority of sarcoidosis patients and helpful in reducing the prednisone dose. The main reason for its limited use is the lack of randomized control trials and evaluation outcomes in a large patient group [12,15].

## Conclusions

Sarcoidosis is a systemic disease that can affect multiple organ systems. Cutaneous inflammation is one of the most common presentations. Providers should be aware of the skin involvement of sarcoidosis, as early recognition and intervention can decrease the risk of complications. The etiology of sarcoidosis is still unknown, and sarcoidal granulomatous reactions due to tattoo pigment have been reported. Systemic sarcoidosis associated with tattoos is rare and cutaneous lesions associated with tattooing are increasingly reported. Therefore, early recognition of skin involvement and intervention can help decrease the risk of complications. With the increased popularity and demand for tattooing, there is a significant need for research related to its complications and treatment. Mycophenolate mofetil is a safe option for treatment, although extensive research and investigation are needed.
